# Comparing neuromodulation targets to reduce cigarette craving and withdrawal: a randomized clinical trial

**DOI:** 10.1038/s41386-025-02106-2

**Published:** 2025-04-25

**Authors:** Nicole Petersen, Michael R. Apostol, Timothy Jordan, Thuc Doan P. Ngo, Nicholas W. Kearley, Edythe D. London, Andrew F. Leuchter

**Affiliations:** 1 Department of Psychiatry and Biobehavioral Sciences, David Geffen School of Medicine at UCLA, Los Angeles, CA USA; 2 Department of Anesthesiology, Emory School of Medicine, Atlanta, GA USA; 3 TMS Clinical and Research Service, Neuromodulation Division, Semel Institute for Neuroscience and Human Behavior at UCLA, Los Angeles, CA USA; 4 Jane and Terry Semel Institute of Neuroscience and Human Behavior, and Department of Psychiatry and Biobehavioral Sciences, University of California at Los Angeles, Los Angeles, CA USA; 5 Department of Molecular and Medical Pharmacology, University of California at Los Angeles, Los Angeles, CA USA

**Keywords:** Addiction, Cognitive neuroscience

## Abstract

Cigarette smoking remains the leading preventable cause of death, emphasizing the need for new therapeutics, such as repetitive transcranial magnetic stimulation (TMS). We tested the hypothesis that TMS to three targets would reduce cigarette craving and withdrawal by modulating connectivity within and between three canonical networks in a randomized clinical trial (ClinicalTrials.gov: NCT03827265). Participants (*N* = 72; DSM-5 tobacco use disorder, ≥1 year of daily smoking) received one session of TMS to hubs of canonical resting-state networks: the dorsolateral prefrontal cortex (dlPFC), superior frontal gyrus (SFG), posterior parietal cortex (PPC), and area v5 (control). Self-reports (craving, withdrawal, and negative affect) and resting-state functional connectivity were measured before and after stimulation. SFG stimulation significantly reduced craving (95% CI, 0.0476–7.9559) and withdrawal (95% CI, 0.9225–8.1063) versus control, with larger effects in men (*D* = 0.59) than in women (*D* = 0.30). SFG stimulation did not change network connectivity, whereas dlPFC stimulation increased somatomotor, default mode, and dorsal attention network connectivity. No severe or unexpected treatment-related adverse events occurred. These findings suggest that SFG shows promise as a target for smoking-cessation treatment, especially for men. Further trials are warranted to confirm efficacy and develop imaging biomarkers for precision neuromodulation.

## Introduction

Cigarette smoking is the leading preventable cause of death worldwide, despite substantial decreases in the prevalence of smoking, and novel therapeutics. The FDA recently cleared deep transcranial magnetic stimulation (TMS) for short-term smoking cessation, recognizing TMS as a safe and effective treatment for Tobacco Use Disorder [1]. However, evidence suggests that conventional figure-8 TMS coils may also be effective, and FDA approval of such a protocol would increase patient access by using TMS devices already available in clinics.

At least ten independent studies have demonstrated that figure-8 TMS reduces cigarette craving [2–11], a predictor of substance use and relapse [12]. Conventional TMS has also led to reductions in heaviness of smoking [2, 3, 8, 11, 13, 14], and improved smoking cessation outcomes [8, 15–17]. Most studies have applied 10–20 Hz stimulation to the left dorsolateral prefrontal cortex (dlPFC), but novel targets revealed by neuroimaging may optimize protocols. Increasingly, such targets appear to be multidimensional network targets rather than unitary locations [18], and even adjacent unitary targets can be stimulated by TMS to influence unique networks [19].

The triple network model of psychopathology (Menon, 2011), implicates Salience Network (SN), Default Mode Network (DMN), and Executive Control Network (ECN) intra- and inter-network connectivity in a variety of dysfunctional brain states. A related theoretical model subsequently proposed that the salience network may act as an attentional “switch” during acute craving, allocating neural resources between the default mode and executive control networks [20, 21]. At least one study found that the magnitude of change in this index correlated with the magnitude of relief from cigarette craving (Lerman et al., 2014), identifying it as an attractive potential biomarker (although subsequent work dampened this enthusiasm, Moradi et al., 2020). Therefore, we sought to test the hypothesis that craving, withdrawal, and negativ e affect may be relieved by stimulating strategic nodes of these networks in a randomized, controlled, crossover clinical trial. We identified surface-level targets in each network accessible with conventional TMS, and hypothesized that stimulating each target would influence (1) craving and withdrawal symptoms, (2) connectivity within the targeted network, and, (3) connectivity between the targeted network and other canonical networks. Our primary objective was to identify the most effective protocol for reducing cigarette craving and withdrawal, and our secondary objective was to investigate the network perturbations produced by stimulating each target. We selected the dlPFC as an executive control network node, the superior frontal gyrus (SFG) as a salience network node, and the posterior parietal cortex (PPC) as a default mode network node.

The targets selected for this clinical trial were chosen because they were (1) close enough to the cortical surface to be accessible with TMS, (2) each a major hub of one of the three networks, and (3) topographically distinct from one another. Other brain regions, such as mPFC, would have also met these criteria, and were not tested only in the interest of parsimony. Left-lateralized targets were selected because left dlPFC was both a hub that met these criteria, and also a well-researched target in its own right. Therefore, we elected to keep all stimulation targets left-lateralized, and all references to dlPFC, SFG, PPC, and v5 throughout the manuscript refer solely to the left hemisphere of each region. In an effort to maximize the stimulation of each network, individualized targets were selected: the voxel within the ROI of interest with peak connectivity to the network of interest was targeted. By stimulating all three targets, we sought to perturb intra- and inter-network dynamics of each member of the triple network model.

Considering sex differences in nicotine withdrawal [20] and neural correlates of craving [21–23], we also planned to determine whether the most effective stimulation target for men and women differed.

## Materials, participants, and methods

The study protocol was approved by the UCLA Institutional Review Board UCLA (Medical IRB 3; #18-000509; initial approval: 07/27/2018). All participants provided informed consent in accordance with the Declaration of Helsinki. The clinical trial protocol is registered on clinicaltrials.gov, NCT03827265.

All analysis code is available at https://github.com/HumanBrainZappingatUCLA/TMS4TUD/.

### II.A. Study design

Data were collected in a repeated measures, crossover design. Participants were asked to arrive on each testing day abstinent from smoking for >12 h. Expired carbon monoxide on each study day was recorded. On each of these days, single-session TMS was delivered. Neuroimaging and behavioral measurements were collected before and immediately after TMS to the three experimental sites (dlPFC, SFG, PPC) and the control site (area v5). The order of stimulation sites was randomized using a random number generator. A graphical overview of study procedures is available in Supplemental Material Fig. S5.

Investigators were not blinded at any stage. Participants could not be blinded to the location of the stimulating magnet, but were not informed which locations were control vs. experimental sites.

### II.B. Participants

Participant demographics are in Table 1. Participants were recruited from the greater Los Angeles community via Craigslist and fliers. All data were collected in the Semel Institute for Neuroscience and Human Behavior at UCLA. A CONSORT diagram is shown in Supplementary Fig. S4.

**Table 1:** Demographic characteristics of 72 included study participants.

**Age**	
*M*	33.3 years
*SD*	6.46 years
Range	21–45 years
**Sex**	
Female	32 (44.4%)
Male	40 (55.6%)
**Gender**	
Female	31 (43.0%)
Male	40 (55.6%)
Prefer not to answer	1 (1.4%)
**Race and ethnicity**	
White, non-hispanic	28 (38.89%)
White, hispanic	7 (9.72%)
Black or African American	17 (23.61%)
Asian	8 (11.11%)
More than One Race	7 (9.72%)
Native Hawaiian or Other Pacific Islander	2 (2.78%)
Unknown or Prefer Not to Specify	3 (4.17%)
**Years of education**	
*M*	14.2 years
*SD*	1.95
Range	12–20 years

Initial eligibility screening was conducted by phone, followed by an in-person session (if qualified). Smoking status was verified through expired carbon monoxide (Micro+ Smokerlyzer®breath CO monitor, Bedfont Scientific Ltd., Maidstone, Kent, UK) and urinary cotinine tests (Abbott™ NicQuick™ Nicotine/Cotinine Test or Accutest, Jant Pharmacal Corp., Encino, CA, USA), and abstinence from other substances was confirmed via urinalysis (Alere Toxicology Services, Portsmouth, VA, USA or Abbott™ iCup™ Zero Exposure Urine Drug Screen) and breathalyzer (Alco-Sensor FST breathalyzer; Intoximeters, Inc.). Comorbid psychiatric disorders were assessed using the Mini International Neuropsychiatric Interview [24]. Participants completed safety questionnaires to ensure eligibility for neuroimaging and neuromodulation. Inclusion criteria required meeting DSM-5 criteria for tobacco use disorder, >1 year of smoking history, daily use >4 cigarettes, and a positive urinary cotinine test. The age range was limited to 18-45 to control for potential age-related interactions with sex differences (e.g., menopause [25–27]).

Participants were excluded if they had a recent (past six months) or current substance use disorder (except mild cannabis use), ongoing psychiatric conditions, major medical conditions, or were pregnant/breastfeeding. Exclusionary psychiatric diagnoses included major depressive disorder, bipolar I/II, anxiety disorders, PTSD, psychotic disorders, eating disorders, and antisocial personality disorder. Exclusion criteria also included medical conditions affecting major organ systems, positive tests for illicit substances (except THC), no biochemical verification of smoking (expired CO < 1 or negative cotinine), MRI/TMS safety risks (e.g., metal implants, seizures), active smoking cessation treatment, or left-hand dominance.

### II.C. Procedures

#### II.C.1. Behavioral measurements

Participants completed the Positive and Negative Affect Schedule (PANAS), Shiffman-Jarvik Withdrawal Questionnaire [28] (SJWS; primary withdrawal measurement), and the Urge to Smoke scale [29] (UTS; primary craving measurement); the latter assessments reliably capture symptoms of craving and withdrawal. All sessions took place in a quiet, controlled, private environment before and after TMS.

#### II.C.2. Brain imaging

Brain imaging data were collected on a 3-Tesla Siemens Prisma Fit MRI scanner with a 32-channel head coil before and immediately after TMS on each test day. A structural T1-weighted scan (TE = 2.24 ms; TR = 2400 ms; isotropic voxels = 0.8 mm^3^) and then functional T2*-weighted multi-band sequence (TE = 37 ms; TR = 800 ms; isotropic voxels = 2 mm^3^, volumes = 588) were collected.

FSL tools were used to apply motion correction, slice-timing correction, and normalization (FEAT, FMRI Expert Analysis Tool). ICA-FIX reduced noise and artifacts. Data were parcellated as in [30], integrating the Schaefer 400-region cortical parcellation [31] with 16 subcortical and 3 cerebellar regions, yielding 419 nodes. Time series were extracted from these parcels, which were assigned to the default mode network, salience network (ventral attention network), and executive control network (frontoparietal network) via the Yeo 7-network solution [32, 33].

#### III.C.3

*TMS*. TMS was administered by a licensed physician at the UCLA Neuromodulation Division. Two devices were used: A Magstim Super Rapid2 Plus1 system equipped with visor2 neuronavigation system (ANT Neuro), and a Magventure Magpro X100 with a Cool-B65 coil with Rogue Research Brainsight neuronavigation. Each stimulation dose was identical: 3000 pulses of 10 Hz stimulation were in 50 pulse trains of 5 s on, 10 s off (total = 15 min). Stimulation intensity was titrated to 100% of motor threshold (MT), with MT determined at the first treatment session as previously described [27, 34, 35].

#### III.C.4

*TMS Targeting:* To personalize TMS targets based on resting-state functional connectivity, we developed a pipeline to identify the voxel within each target region (dlPFC, SFG, PPC, and v5) with maximum connectivity to key brain networks (ECN, DMN, SN, and visual). Preprocessed resting-state data were decomposed into 20 independent components using FSL’s MELODIC. Network hubs (posterior cingulate for default mode, inferior frontal gyrus for executive control, insula for salience, and area v5 for visual) were used to assign each component to a specific network. The TMS target was the voxel within each ROI with peak connectivity to the respective network. That is, the dlPFC, SFG, PPC, and v5 ROIs were defined and masked a priori, and masks are available for further review in our GitHub repository: https://github.com/HumanBrainZappingatUCLA/TMS4TUD/tree/main/ROIs. The dlPFC was selected as a hub of the executive control network, so the voxel within the dlPFC mask with peak connectivity to the executive control network was targeted. The SFG was selected as a hub of the salience network, so the voxel within the SFG mask with peak connectivity to the salience network was targeted. The PPC was selected as a hub of the default mode network, so the voxel within the PPC mask with peak connectivity to the default mode network was selected. Area v5 was selected as a control, but nevertheless we applied the same network-guided mapping and selected the voxel within that area with peak connectivity to the visual cortex to target with TMS. This resulted in stimulation coordinates clustered throughout each stimulation target. The distribution of these stimulation targets is shown in Supplementary Fig. S2.

*II.D. Outcome measures and statistical analysis:* Self-reported cigarette craving, withdrawal, and negative affect during abstinence were measured using the Urge to Smoke (UTS) scale, Shiffman-Jarvik Withdrawal scale (SJWS), and the negative affect subscale of the Positive and Negative Affect Schedule (PANAS-), each administered before and after each TMS session. Linear mixed models (LMMs) were used to estimate the effect of TMS on each behavioral outcome using the smf.mixedlm function from the *statsmodels* library in Python, with the Restricted Maximum Likelihood (REML) method for model fitting. For each measure (UTS, SJWS, PANAS-), a separate LMM was fit to compare behavioral responses after stimulation at each experimental site (dlPFC, SFG, PPC) versus the control site (the v5 region of visual cortex). Each model included fixed effects for the time point (Pre/Post) and target group (Experimental/Control), as well as their interaction, and random intercepts for participants to account for within-subject correlations. The effect of sex on each primary outcome measurement was tested using a LMM, with participants entered as a random effect and sex entered as a fixed effect. All baseline (pre-TMS) data were included in the model.

#### II.E. Brain imaging analysis

To analyze the effects of stimulating each neural target on functional connectivity, we calculated within- and between-network connectivity (using the Schaefer parcellation and Yeo 7-network solution), initially focusing on the DMN, SN, and ECN [32, 33]. Connectivity values for network pairs were computed and averaged, then entered into difference scores. All statistics were carried out in Python (version 3.9.6) with the libraries *numpy*, *pandas*, *scipy*, and *statsmodels* implemented as needed. Paired *t*-tests compared pre- and post-session connectivity for each network pair, grouped by target. Next, LMMs were fitted in to model average connectivity as a function of time (pre/post, fixed effect) and target (fixed effect) while accounting for variability between participants (random effects). This was applied to the networks named above, for which we had a priori hypotheses, and also the remaining networks in the parcellation (limbic, dorsal attention, subcortical, visual, and somatomotor networks). Because all possible network pairs were included in the analysis, a familywise error correction was applied (false discovery rate using the Benjamini-Hochberg procedure).

#### II.F. Role of the funding source

The funder of the study had no role in study design, data collection, data analysis, data interpretation, or writing of the report.

## Results

The trial’s planned stopping point was *N* = 60 based on an a priori power analysis (effect size estimate derived from unpublished data); 171 individuals were enrolled into the trial, and the total number of sessions for each target were:

dlPFC, *N* = 61

SFG, *N* = 60

PPC, *N* = 62

v5, *N* = 66

Not all participants who enrolled completed all data collection sessions, and reasons for not completing are given in the CONSORT diagram. Fifty-seven people completed all four data collection sessions. Due to the random pattern of missing data, participants who dropped out after 1, 2, or 3 sessions may have completed stimulation to any target, and therefore data are available for *N* ≥ 60 individuals for each data collection session (dlPFC, SFG, PPC, and v5).

Demographics are given in Table 1.

**Fig. 1:**
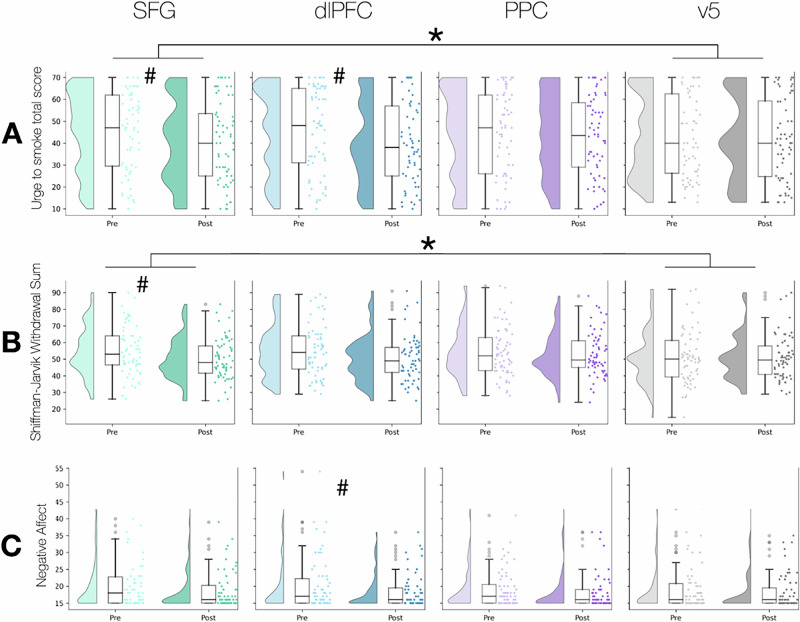
Effects of TMS on craving, withdrawal, and negative affect. Raincloud plots depict the change in Urge to Smoke scale scores (Panel **A**), Shiffman-Jarvik Withdrawal scale scores (Panel **B**), and Negative Affect scores (Panel **C**) from pre- to post-stimulation for the four stimulation targets. Each plot combines a density curve (left) indicating the distribution of scores, individual data points (middle) showing individual scores for pre- and post-stimulation conditions, and boxplots (right) summarizing the median and spread of the data. *Symbols:* Asterisk (*) denotes a significant interaction, indicating a significantly greater change in the experimental condition compared to the control. Hashtag (^#^) denotes a significant main effect of time (i.e., significant change from pre- to post-stimulation). The site-by-time interaction was significant for the effect of TMS to SFG vs. control (*p* = 0.047) on craving and withdrawal (*p* = 0.01). The main effect of time on craving was significant for the SFG (*p* = 0.0013) and dlPFC (*p* = 0.0053) conditions. The main effect of time on withdrawal was significant for the SFG (*p* = 0.0012) and dlPFC (*p* = 0.0016). The main effect of time on negative affect was significant for the dlPFC only (*p* = 0.0016).

Our primary outcomes were the effects of stimulation on craving, withdrawal, and negative affect. A significant time-by-site interaction was found for stimulation to the SFG on craving (β = 4.00, 95% CI, 0.08-7.96, SE = 2.02, t = 1.98, p = 0.047) but not for dlPFC vs. control (β = 3.56, SE = 2.24, t = 1.59, p = 0.11) or PPC vs. control (β = 0.977, SE = 2.24, t = 0.44, p = 0.66) (Fig. 1A).

A significant time-by-site interaction was found for stimulation to the SFG on withdrawal (*β* = 4.51, 95% CI, 0.92–8.11, SE = 1.83, *t* = 2.46, *p* = 0.01), but not for dlPFC vs. control (*β* = 3.42, SE = 1.86, *t* = 1.83, *p* = 0.07) or PPC vs. control (*β* = 1.47, SE = 1.92, *t* = 0.77, *p* = 0.44) (Fig. 1B).

No significant main effects on negative affect or time-by-target interactions on negative affect were found as a result of stimulation delivered to any target (Fig. 1C) (dlPFC vs. control: *β* = 0.56, SE = 0.99, *t* = 0.567, *p* = 0.57; SFG vs. control*: β* = 0.65, SE = 1.17, *t* = 0.56, *p* = 0.58; PPC vs. control*: β* = −0.25, SE = 0.95, *t* = −0.27, *p* = 0.79).

Exploratory tests for *main* (rather than site-by-time interaction) effects of stimulation indicated that TMS to dlPFC reduced craving (11.64% decrease, *p* = 0.0053), as did SFG TMS (10.69% decrease, *p* = 0.0013), but not v5 (1.97% decrease, *p* = 0.47) nor PPC (4.10% reduction, *p* = 0.186). A similar pattern was observed for effects on withdrawal: Withdrawal was significantly lower after TMS to dlPFC (*p* = 0.0016) and SFG (*p* = 0.0012), but not PPC (*p* = 0.2059) or v5 (*p* = 0.9736). Negative affect was significantly lower after TMS to dlPFC (*p* = 0.0016) but not SFG (*p* = 0.0823), PPC (*p* = 0.1419), or v5 (*p* = 0.0550).

**Fig. 2:**
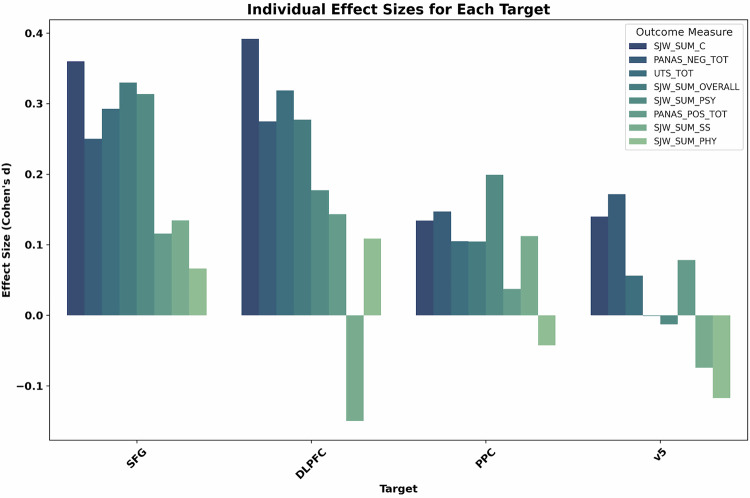
Effect sizes showing TMS’ effect on each inventory’s subscales, separated by stimulation target. Stimulation to SFG and dlPFC produced some medium-sized effects, whereas stimulation to PPC and v5 led to no or small effects.

Although not a primary outcome, we also explored the effect of each stimulation type on unused subscales of primary analysis instruments (Shiffman-Jarvik Withdrawal Scale: Craving, Psychological Withdrawal, Physiological Withdrawal, Stimulation/Sedation, and Appetite; PANAS: positive affect); results are shown in Fig. 2. The largest effects were craving reductions from SFG and dlPFC stimulation, followed by reductions in withdrawal as a result of SFG stimulation.

Planned imaging analyses (secondary outcome) focused on the default mode, executive, and salience networks. Connectivity within and between these networks was not significantly different after TMS to any target, nor did the time-by-target interaction, all *p*s > 0.05.

Safety outcomes are detailed in Supplementary Table S1. Two participants withdrew from the study due to discomfort from the stimulation procedures, both on days randomized to dlPFC stimulation. There were no study-related severe or unexpected adverse events.

Potential confounds were evaluated. Fagerström Test for Nicotine Dependence (FTND) scores were related to craving (*p* = 0.0003), withdrawal (*p* < 0.0001), and negative affect (*p* = 0.018), but did not interact significantly with the effect of time on any variable (all interaction *p*s > 0.24). Age was unrelated to dependent variables (*p*s > 0.10). Years of education was related to baseline psychological withdrawal, *p*_uncorrected_ = 0.04. Therefore, these variables were excluded from subsequent models.

**Fig. 3:**
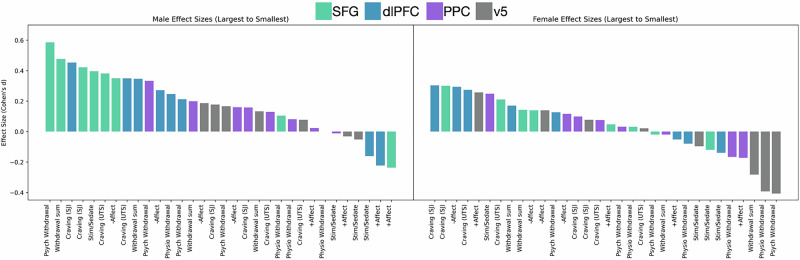
Effect sizes of each stimulation type separated by sex. The pattern of effect sizes appears different for men and women. Men show a small-medium change (reduction) in craving and withdrawal as a response to TMS to the SFG and dlPFC. The largest effect in the trial is a reduction in psychological withdrawal resulting from SFG stimulation in men only. Women show smaller effects overall, especially with respect to reductions in psychological withdrawal.

Men and women did not differ significantly in craving (*p* = 0.49), withdrawal (*p* = 0.15), or negative affect (*p* = 0.32). Three-way interactions between sex, target, and time on craving, withdrawal, and negative affect were not significant (*p*s > 0.05). However, the time-by-sex interaction on overall withdrawal was significant for SFG stimulation only (*p* = 0.04). Disaggregating effect sizes by sex, SFG stimulation had larger effects in men than women (Fig. 3). The largest effect in the dataset for men was approximately twice as large as the largest effect size for women.

Stability of baseline measurements and adequacy of the control condition were evaluated. Baseline (pre-TMS) measurements of craving (UTS), withdrawal (SJWS), and negative affect (PANAS-) were stable across the four days of testing sessions (ps = 0.85, 0.89, and 0.79, respectively), adding confidence that the washout period was adequate, and randomization successful.

Withdrawal (*β* = −0.039, SE = 1.193, *z* = −0.03, *p* = 0.97) and craving (*β* = 0.84, SE = 1.156, *z* = 0.727, *p* = 0.467) did not change significantly or marginally from TMS delivered to v5 (control target). Negative affect also did not change significantly from TMS to this target (*β* = 1.246, SE = 0.649, *z* = 1.919, *p* = 0.055), a strong trend was observed. *Post hoc* testing of individual PANAS- items showed that this effect was primarily driven by reduction in ratings of the “nervous” item (*p* = 0.0014) after stimulation.

Network connectivity was also similar at baseline between sessions. Within-network connectivity of limbic, dorsal attention, executive control, subcortical, visual, salience, somatomotor, and default mode networks was calculated and compared between test days. Comparing the connectivity of each baseline network-network pair by ANOVA did not yield any significant differences, *p*s ranging from 0.35 (salience-salience connectivity) to 0.996 (dorsal attention network-limbic network connectivity. Baseline network connectivity is shown in Supplementary Fig. S1.

**Fig. 4:**
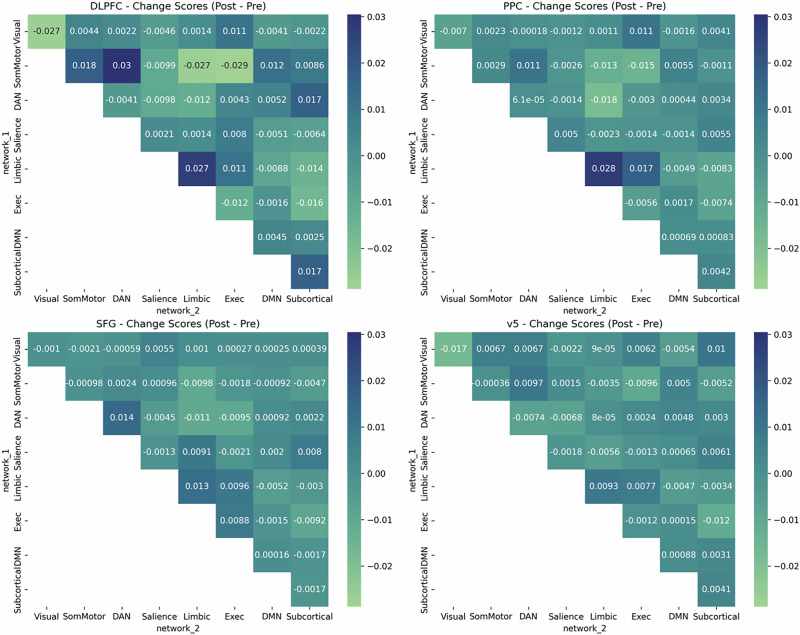
Effects of TMS on canonical network connectivity. Only TMS to dlPFC produced changes in network connectivity that survived familywise error correction. These changes included both increases in connectivity (between the somatomotor network and the default mode network, and between the somatomotor network and dorsal attention network), and decreases in connectivity (within the visual network, and between the somatomotor network and executive control network).

Baseline functional connectivity differed between men and women (Supplementary Table 2 and Supplementary Fig. S3). Exploratory analysis of all networks revealed that dlPFC stimulation only changed network connectivity (Fig. 4). After dlPFC TMS, connectivity was significantly higher:

1. Between the somatomotor network and default mode network (*p*_FDR_ = 0.03).
2. Between the somatomotor network and dorsal attention network (*p*_FDR_ = 0.02).

After dlPFC TMS, connectivity was significantly lower:

1. Within the visual network (*p*_FDR_ = 0.02).
2. Between the somatomotor network and executive network (*p*_FDR_ = 0.01).

## Discussion

In this randomized crossover trial evaluating the acute effects of single-session TMS delivered to three experimental sites (dlPFC, SFG, PPC) and a control site, stimulation to the SFG significantly reduced both craving and withdrawal in individuals with Tobacco Use Disorder compared to the control target, replicating earlier findings [9]. Negative affect was not significantly impacted by stimulation to any target as compared to the control target. Our findings are also consistent with previous evidence that stimulation to the dlPFC reduces craving [2–4, 6, 8, 10, 11]. The effect sizes for men were larger than the effect sizes for women. SFG is a promising target for smoking cessation trials and can be implemented using standard TMS equipment.

We selected stimulation targets that are hubs of resting-state networks (dlPFC for executive control, SFG for salience, PPC for default mode, and v5 for visual), expecting network-specific effects. However, dlPFC was globally influential, inducing within- and between-network changes in several networks, including networks that we did not expect to be modulated by TMS. By contrast, SFG and v5 produced no significant network-level changes. Nonetheless, SFG stimulation effectively reduced craving and withdrawal, suggesting alternative neural mechanisms. Together, these findings do not support the triple network model as a framework for understanding craving reduction. Future research is needed to search for circuits that do underlie the craving reductions observed, and incorporate them into novel circuit-guided studies that do not rely on the triple-network model. Circuit-based approaches have already advanced TMS treatments for depression, where a specific circuit – connectivity between dlPFC and the subgenual anterior cingulate cortex circuits – is related to treatment response [36–40]. Analyses to uncover specific craving relief circuits could focus on brain regions known to be involved in TUD, such as the mesocorticolimbic circuit and insula, and explore how connectivity changes within and between these regions corresponds to the behavioral results observed here. Data-driven metrics capable of minimizing the many dimensions inherent in fMRI analyses, such as principal component analysis and graph theory metrics, may also be useful for these exploratory investigations. Symptom-specific targeting is also emerging for depression treatment [41], and our finding that craving and withdrawal may respond to different targets suggests that this is a potential avenue for addiction treatment as well.

Some important limitations influence the interpretation of results. This trial was impacted by the COVID-19 pandemic, with data collection spanning from March 2019 to August 2024. During this time, changes such as increased use of electronic nicotine delivery systems and cannabis may have influenced results. Additionally, our exclusion of participants with comorbid psychiatric and substance use disorders limits generalizability, as TMS may be effective in treating such patients [42]. The trial was also underpowered to detect sex differences, likely due to the higher demands of detecting ordinal interactions [43]. Although treatment protocols were the same, it is possible that the use of two different devices may have influenced the results. Additionally, the use of a single stimulation frequency is a limitation, as area-dependent frequency effects have been reported in other TMS studies [44, 45]. It is plausible, if not likely, that location and frequency interact, such that the optimal frequency to influence brain networks and behavior depends on the location stimulated. Finally, the relatively low dose of neuromodulation may have limited the separation between experimental and control conditions, as higher doses are typically used in treatment protocols.

Consideration should also be given to nomenclature, both with respect to the networks and brain targets involved in this investigation. The brain regions and networks involved are all known by other names, and controversies remain in the differentiation and specification of each (for a discussion of network nomenclature controversies and a possible resolution, see refs. [46, 47]. With respect to the brain regions stimulated, each active target is described anatomically, but this not a strict definition, and all targets span distinct functionally-defined nodes and cytoarchitechtonic areas. The SFG target could just as accurately be called the left dorsomedial prefrontal cortex, and the part of the SFG stimulated in this study ostensibly overlaps with the medial prefrontal cortical area that is also stimulated by the deep TMS H7 coil. Notably, this device has shown promise in treating both alcohol use disorder [48] and methamphetamine use disorder [49]. Similarly, the PPC region stimulated in this experiment is large, and spans many distinct regions that are functionally and anatomically distinct. Stimulation delivered to different areas within this region of interest may produce different results, and further atomization may be more informative in future studies. Whether better clinical results could be generated by stimulating more specific parts of the PPC, other nodes of the DMN, or any of these using frequencies that are more aligned with intrinsic cortical oscillations, remains to be seen.

Personalized targeting as a component of this study. However, it should be noted that this approach cannot be compared to non-personalized targeting in this investigation. Investigations comparing the two methods have been concentrated in investigations of TMS for the treatment of mood disorders, and the advantages of personalized targeting, which is more burdensome than heuristic targeting, has not been conclusively demonstrated. Although some studies have reported improved clinical outcomes in patients receiving personalized targeting [39] and this is thought to be one of the mechanisms by which novel protocols improve outcomes [50], direct investigations have shown only small benefits of personalization [51]. The results reported here cannot provide evidence for or against personalized targeting.

Phase 2 and 3 trials are needed to confirm the efficacy of SFG stimulation for smoking cessation, followed by optimization trials examining factors such as stimulation frequency, dosing, spacing, and neural context. Additionally, future studies should compare the tolerability of different TMS protocols. Tailoring addiction treatment based on individual symptom profiles could also be explored, with different neural targets emerging for individuals whose relapse risk is driven by craving, withdrawal, or negative affect. Investigating symptom-specific neural targets may help overcome observed sex differences and other individual-level factors that influence brain circuits related to craving and withdrawal relief. The costs and benefits of using individualized targeting, as was employed in this study, also warrant empirical investigation.

## Supplementary information

Supplemental materials

## Data Availability

De-identified data will be made publicly available once the investigators’ currently-planned analyses are complete and disseminated.

## References

[1] Zangen A, Moshe H, Martinez D, Barnea-Ygael N, Vapnik T, Bystritsky A, et al. Repetitive transcranial magnetic stimulation for smoking cessation: a pivotal multicenter double-blind randomized controlled trial. World Psychiatry. 2021;20:397–404. 34505368 10.1002/wps.20905 PMC8429333

[2] Abdelrahman AA, Noaman M, Fawzy M, Moheb A, Karim AA, Khedr EM. A double-blind randomized clinical trial of high frequency rTMS over the DLPFC on nicotine dependence, anxiety and depression. Sci Rep. 2021;11:1640. 10.1038/s41598-020-80927-5 PMC7810712 33452340

[3] Amiaz R, Levy D, Vainiger D, Grunhaus L, Zangen A. Repeated high‐frequency transcranial magnetic stimulation over the dorsolateral prefrontal cortex reduces cigarette craving and consumption. Addiction 2009;104:653–60. 19183128 10.1111/j.1360-0443.2008.02448.x

[4] Chang D, Zhang J, Peng W, Shen Z, Gao X, Du Y, et al. Smoking cessation with 20 Hz repetitive transcranial magnetic stimulation (rTMS) applied to two brain regions: a pilot study. Front Hum Neurosci. 2018;12:344. 30319373 10.3389/fnhum.2018.00344 PMC6166007

[5] Flores-Leal M, Sacristán-Rock E, Jiménez-Angeles L, Azpiroz-Leehan J. Low frequency repetitive transcranial magnetic stimulation effects over dorsolateral prefrontal cortex in moderate nicotine dependent subjects. In: VI Latin American Congress on Biomedical Engineering CLAIB 2014. Paraná, Argentina: Springer; 2015. p. 317–20.

[6] Johann M, Wiegand R, Kharraz A, Bobbe G, Sommer G, Hajak G, et al. Transcranial magnetic stimulation for nicotine dependence. Psychiatrische Prax. 2003;30:S129–31. 14509058

[7] Li X, Hartwell KJ, Owens M, LeMatty T, Borckardt JJ, Hanlon CA, et al. Repetitive Transcranial Magnetic Stimulation of the Dorsolateral Prefrontal Cortex Reduces Nicotine Cue Craving. Biol Psychiatry. 2013;73:714–20. 23485014 10.1016/j.biopsych.2013.01.003 PMC3615051

[8] Li X, Hartwell KJ, Henderson S, Badran BW, Brady KT, George MS. Two weeks of image-guided left dorsolateral prefrontal cortex repetitive transcranial magnetic stimulation improves smoking cessation: A double-blind, sham-controlled, randomized clinical trial. Brain Stimul. 2020;13:1271–9. 32534252 10.1016/j.brs.2020.06.007 PMC7494651

[9] Rose JE, McClernon FJ, Froeliger B, Behm FM, Preud’homme X, Krystal AD. Repetitive Transcranial Magnetic Stimulation of the Superior Frontal Gyrus Modulates Craving for Cigarettes. Biol Psychiatry. 2011;70:794–9. 21762878 10.1016/j.biopsych.2011.05.031

[10] Shevorykin A, Carl E, Mahoney MC, Hanlon CA, Liskiewicz A, Rivard C, et al. Transcranial Magnetic Stimulation for Long-Term Smoking Cessation: Preliminary Examination of Delay Discounting as a Therapeutic Target and the Effects of Intensity and Duration. Front Hum Neurosci. 2022;16:920383. 10.3389/fnhum.2022.920383 PMC9300313 35874156

[11] Upton S, Brown AA, Ithman M, Newman-Norlund R, Sahlem G, Prisciandaro JJ, et al. Effects of Hyperdirect Pathway Theta Burst Transcranial Magnetic Stimulation on Inhibitory Control, Craving, and Smoking in Adults With Nicotine Dependence: A Double-Blind, Randomized Crossover Trial. Biol Psychiatry. 2023;8:1156–65. 10.1016/j.bpsc.2023.07.014 PMC10840958 37567363

[12] Vafaie N, Kober H. Association of Drug Cues and Craving With Drug Use and Relapse: A Systematic Review and Meta-analysis. JAMA Psychiatry. 2022;79:641–50. 35648415 10.1001/jamapsychiatry.2022.1240 PMC9161117

[13] Eichhammer P, Johann M, Kharraz A, Binder H, Pittrow D, Wodarz N, et al. High-frequency repetitive transcranial magnetic stimulation decreases cigarette smoking. J Clin Psychiatry. 2003;64:951–3. 12927012 10.4088/jcp.v64n0815

[14] Prikryl R, Ustohal L, Kucerova HP, Kasparek T, Jarkovsky J, Hublova V, et al. Repetitive transcranial magnetic stimulation reduces cigarette consumption in schizophrenia patients. Prog Neuro Psychopharmacol Biol Psychiatry. 2014;49:30–5. 10.1016/j.pnpbp.2013.10.019 24211840

[15] Dieler AC, Dresler T, Joachim K, Deckert J, Herrmann MJ, Fallgatter AJ. Can intermittent theta burst stimulation as add-on to psychotherapy improve nicotine abstinence? Results from a pilot study. Eur Addiction Res. 2014;20:248–53. 10.1159/000357941 24924851

[16] Sheffer CE, Bickel WK, Brandon TH, Franck CT, Deen D, Panissidi L, et al. Preventing relapse to smoking with transcranial magnetic stimulation: Feasibility and potential efficacy. Drug Alcohol Depend. 2018;182:8–18. 29120861 10.1016/j.drugalcdep.2017.09.037 PMC5836507

[17] Trojak B, Meille V, Achab S, Lalanne L, Poquet H, Ponavoy E, et al. Transcranial magnetic stimulation combined with nicotine replacement therapy for smoking cessation: a randomized controlled trial. Brain Stimul. 2015;8:1168–74. 26590478 10.1016/j.brs.2015.06.004

[18] Noble S, Curtiss J, Pessoa L, Scheinost D. The tip of the iceberg: A call to embrace anti-localizationism in human neuroscience research. Imaging Neurosci. 2024;2:1–10. 10.1162/imag_a_00138 PMC12247580 40740614

[19] Eldaief MC, McMains S, Izquierdo-Garcia D, Daneshzand M, Nummenmaa A, Braga RM. Network-specific metabolic and haemodynamic effects elicited by non-invasive brain stimulation. Nature Mental Health. 2023;1:346–60. 10.1038/s44220-023-00046-8 PMC10655825 37982031

[20] Faulkner P, Petersen N, Ghahremani DG, Cox CM, Tyndale RF, Hellemann GS, et al. Sex differences in tobacco withdrawal and responses to smoking reduced-nicotine cigarettes in young smokers. Psychopharmacology. 2018;235:193–202. 29022071 10.1007/s00213-017-4755-x PMC6726112

[21] Dumais KM, Franklin TR, Jagannathan K, Hager N, Gawrysiak M, Betts J, et al. Multi-site exploration of sex differences in brain reactivity to smoking cues: Consensus across sites and methodologies. Drug Alcohol Depend. 2017;178:469–76. 28711813 10.1016/j.drugalcdep.2017.05.044 PMC5567981

[22] Perez Diaz M, Pochon JB, Ghahremani DG, Dean AC, Faulkner P, Petersen N, et al. Sex Differences in the Association of Cigarette Craving With Insula Structure. Int J Neuropsychopharmacol. 2021;24:624–33. 33830218 10.1093/ijnp/pyab015 PMC8378076

[23] Wetherill RR, Young KA, Jagannathan K, Shin J, O’Brien CP, Childress AR, et al. The impact of sex on brain responses to smoking cues: a perfusion fMRI study. Biol Sex Differences 2013;4:9. 10.1186/2042-6410-4-9 PMC3643879 23628003

[24] Sheehan DV, Lecrubier Y, Sheehan KH, Amorim P, Janavs J, Weiller E, et al. The Mini-International Neuropsychiatric Interview (M.I.N.I.): the development and validation of a structured diagnostic psychiatric interview for DSM-IV and ICD-10. J Clin Psychiatry. 1998;59:22–33. 9881538

[25] Kryatova MS, Seiner SJ, Brown JC, Siddiqi SH. Older age associated with better antidepressant response to H1-coil transcranial magnetic stimulation in female patients. J Affect Disord. 2024;351:66–73. 38244806 10.1016/j.jad.2024.01.160

[26] Sackeim HA, Aaronson ST, Carpenter LL, Hutton TM, Mina M, Pages K, et al. Clinical outcomes in a large registry of patients with major depressive disorder treated with Transcranial Magnetic Stimulation. J Affect Disord. 2020;277:65–74. 32799106 10.1016/j.jad.2020.08.005

[27] Slan AR, Citrenbaum C, Corlier J, Ngo D, Vince-Cruz N, Jackson NJ, et al. The role of sex and age in the differential efficacy of 10 Hz and intermittent theta-burst (iTBS) repetitive transcranial magnetic stimulation (rTMS) treatment of major depressive disorder (MDD). J Affect Disord. 2024;366:106–12. 39187197 10.1016/j.jad.2024.08.129

[28] Shiffman SM, Jarvik ME. Smoking withdrawal symptoms in two weeks of abstinence. Psychopharmacology. 1976;50:35–9. 827760 10.1007/BF00634151

[29] Jarvik ME, Madsen DC, Olmstead RE, Iwamoto-Schaap PN, Elins JL, Benowitz NL. Nicotine blood levels and subjective craving for cigarettes. Pharmacol Biochem Behav. 2000;66:553–8. 10899369 10.1016/s0091-3057(00)00261-6

[30] Van De Ville D, Farouj Y, Preti MG, Liégeois R, Amico E. When makes you unique: Temporality of the human brain fingerprint. Sci Adv. 2021;7:eabj0751. 34652937 10.1126/sciadv.abj0751 PMC8519575

[31] Schaefer A, Kong R, Gordon EM, Laumann TO, Zuo XN, Holmes AJ, et al. Local-global parcellation of the human cerebral cortex from intrinsic functional connectivity MRI. Cereb Cortex. 2018;28:3095–114. 28981612 10.1093/cercor/bhx179 PMC6095216

[32] Yeo BT, Krienen FM, Sepulcre J, Sabuncu MR, Lashkari D, Hollinshead M, et al. The organization of the human cerebral cortex estimated by intrinsic functional connectivity. J Neurophysiol. 2011;106:1125–65. 10.1152/jn.00338.2011 PMC3174820 21653723

[33] Yeo BTT, Krienen FM, Chee MWL, Buckner RL. Estimates of segregation and overlap of functional connectivity networks in the human cerebral cortex. NeuroImage 2014;88:212–27. 24185018 10.1016/j.neuroimage.2013.10.046 PMC4007373

[34] Chu SA, Tadayonnejad R, Corlier J, Wilson AC, Citrenbaum C, Leuchter AF. Rumination symptoms in treatment-resistant major depressive disorder, and outcomes of repetitive Transcranial Magnetic Stimulation (rTMS) treatment. Translational. Psychiatry 2023;13:293. 10.1038/s41398-023-02566-4 PMC10491586 37684229

[35] Citrenbaum C, Corlier J, Ngo D, Vince-Cruz N, Wilson A, Wilke SA, et al. Pretreatment pupillary reactivity is associated with differential early response to 10 Hz and intermittent theta-burst repetitive transcranial magnetic stimulation (rTMS) treatment of major depressive disorder (MDD). Brain Stimul. 2023;16:1566–71. 37863389 10.1016/j.brs.2023.10.006

[36] Cash RFH, Cocchi L, Lv J, Fitzgerald PB, Zalesky A. Functional Magnetic Resonance Imaging–Guided Personalization of Transcranial Magnetic Stimulation Treatment for Depression. JAMA Psychiatry. 2021;78:337–9. 33237320 10.1001/jamapsychiatry.2020.3794 PMC7689561

[37] Fox MD, Buckner RL, White MP, Greicius MD, Pascual-Leone A. Efficacy of Transcranial Magnetic Stimulation Targets for Depression Is Related to Intrinsic Functional Connectivity with the Subgenual Cingulate. Biol Psychiatry. 2012;72:595–603. 22658708 10.1016/j.biopsych.2012.04.028 PMC4120275

[38] Kong G, Wei L, Wang J, Zhu C, Tang Y. The therapeutic potential of personalized connectivity-guided transcranial magnetic stimulation target over group-average target for depression. Brain Stimul. 2022;15:1063–4. 35931377 10.1016/j.brs.2022.07.054

[39] Oathes DJ, Gonzalez AF, Grier J, Blaine C, Garcia SD, Linn KA. Clinical Response to fMRI-guided Compared to Non-Image Guided rTMS in Depression and PTSD: A Randomized Trial. medRxiv. 10.1101/2024.07.29.24311191.

[40] Siddiqi SH, Weigand A, Pascual-Leone A, Fox MD. Identification of personalized transcranial magnetic stimulation targets based on subgenual cingulate connectivity: an independent replication. Biol Psychiatry. 2021;90:e55–6. 33820629 10.1016/j.biopsych.2021.02.015

[41] Siddiqi SH, Taylor SF, Cooke D, Pascual-Leone A, George MS, Fox MD. Distinct Symptom-Specific Treatment Targets for Circuit-Based Neuromodulation. AJP. 2020;177:435–46. 10.1176/appi.ajp.2019.19090915 PMC8396109 32160765

[42] Blyth SH, Zabik NL, Krosche A, Prisciandaro JJ, Ward HB. rTMS for Co-occurring Psychiatric and Substance Use Disorders: Narrative Review and Future Directions. Curr Addiction Rep. 2024;11:342–51. 10.1007/s40429-024-00542-6 PMC12306425 40735243

[43] Strube MJ, Bobko P. Testing hypotheses about ordinal interactions: Simulations and further comments. J Appl Psychol. 1989;74:247.

[44] Romei V, Gross J, Thut G. On the role of prestimulus alpha rhythms over occipito-parietal areas in visual input regulation: correlation or causation? J Neurosci. 2010;30:8692–7. 20573914 10.1523/JNEUROSCI.0160-10.2010 PMC6634639

[45] Okazaki YO, Nakagawa Y, Mizuno Y, Hanakawa T, Kitajo K. Frequency- and Area-Specific Phase Entrainment of Intrinsic Cortical Oscillations by Repetitive Transcranial Magnetic Stimulation. Front Hum Neurosci. 2021;15:608947. 10.3389/fnhum.2021.608947 PMC7994763 33776666

[46] Uddin LQ, Betzel RF, Cohen JR, Damoiseaux JS, De Brigard F, Eickhoff SB, et al. Controversies and progress on standardization of large-scale brain network nomenclature. Netw Neurosci. 2023;7:864–905. 37781138 10.1162/netn_a_00323 PMC10473266

[47] Kong R, Spreng RN, Xue A, Betzel RF, Cohen JR, Damoiseaux JS, et al. A network correspondence toolbox for quantitative evaluation of novel neuroimaging results. Nat Commun. 2025;16:2930. 10.1038/s41467-025-58176-9 PMC11937327 40133295

[48] Harel M, Perini I, Kämpe R, Alyagon U, Shalev H, Besser I, et al. Repetitive transcranial magnetic stimulation in alcohol dependence: a randomized, double-blind, sham-controlled proof-of-concept trial targeting the medial prefrontal and anterior cingulate cortices. Biol Psychiatry. 2022;91:1061–9. 35067356 10.1016/j.biopsych.2021.11.020

[49] Zhao D, Zeng N, Zhang HB, Zhang Y, Shan J, Luo H, et al. Deep magnetic stimulation targeting the medial prefrontal and anterior cingulate cortices for methamphetamine use disorder: a randomised, double-blind, sham-controlled study. Gen Psychiatry. 2023;36:e101149. 10.1136/gpsych-2023-101149 PMC10533780 37781340

[50] Cole EJ, Stimpson KH, Bentzley BS, Gulser M, Cherian K, Tischler C, et al. Stanford accelerated intelligent neuromodulation therapy for treatment-resistant depression. Am J Psychiatry. 2020;177:716–26. 32252538 10.1176/appi.ajp.2019.19070720

[51] Elbau IG, Lynch CJ, Downar J, Vila-Rodriguez F, Power JD, Solomonov N, et al. Functional connectivity mapping for rTMS target selection in depression. Am J Psychiatry. 2023;180:230–40. 36855880 10.1176/appi.ajp.20220306 PMC11446248

